# *Puya ukumari* sp. nov. (Bromeliaceae, Puyoideae), a new montane bromeliad species from southeastern Peru

**DOI:** 10.3897/phytokeys.280.211255

**Published:** 2026-10-15

**Authors:** Jhon A. Yuca-Palomino, Norma G. Mamani-Mayta, Luis A. Champi-Charalla, Elias Condori-Cuti, Narciso Llacta-Gomez, Italo F. Treviño-Zevallos, Eric J. Gouda, Adrian Forsyth, Andrew Whitworth, Ruthmery Pillco-Huarcaya

**Affiliations:** 1 Asociación para la Conservación de la Cuenca Amazónica (ACCA), Cusco, Peru Asociación para la Conservación de la Cuenca Amazónica (ACCA) Cusco Peru; 2 Universidad Nacional de San Antonio Abad del Cusco (UNSAAC), Cusco, Peru Universidad Nacional de San Antonio Abad del Cusco (UNSAAC) Cusco Peru; 3 Comunidad Campesina Juan Velasco Alvarado, Paucartambo, Cusco, Peru Comunidad Campesina Juan Velasco Alvarado Paucartambo Peru; 4 Universidad Tecnológica del Perú (UTP), Tacna y Arica 160, 04001, Arequipa, Peru Universidad Tecnológica del Perú (UTP) Arequipa Peru; 5 Quantitative Biodiversity Dynamics (QBD), Utrecht University (UU), Utrecht, Netherlands Quantitative Biodiversity Dynamics (QBD), Utrecht University (UU) Utrecht Netherlands; 6 Botanic Garden, Utrecht University (UU), Utrecht, Netherlands Botanic Garden, Utrecht University (UU) Utrecht Netherlands; 7 Andes Amazon Fund, 1759 1/2, R St NW #200, 20009, Washington DC, USA Andes Amazon Fund Washington DC United States of America; 8 Climate Corridors, Washington DC, USA Climate Corridors Washington DC United States of America; 9 Department of Biology and Sabin Center for Environment and Sustainability, Wake Forest University, Winston-Salem, NC, USA Department of Biology and Sabin Center for Environment and Sustainability, Wake Forest University Winston-Salem United States of America; 10 School of Animal, Rural and Environmental Sciences, Nottingham Trent University, Nottingham, UK School of Animal, Rural and Environmental Sciences, Nottingham Trent University Nottingham United Kingdom

**Keywords:** Bromeliaceae, montane forest, Peru, plant taxonomy, Tropical Andes, Bromeliaceae, bosque montano, Perú, taxonomía vegetal, Andes Tropicales

## Abstract

Montane forests of the Tropical Andes are among the most diverse and endemic-rich ecosystems in the world; however, they remain insufficiently documented, particularly in remote and topographically complex landscapes. Here, we describe *Puya ukumari***sp. nov**. (Bromeliaceae), a new species from humid montane forests and forest–puna ecotonal habitats in the Kosñipata and Mapacho valleys of southeastern Peru, occurring at elevations between 2,300 and 3,300 m a.s.l. The species was first detected during a long-term ecological monitoring program in the region but remained unrecognized for several years due to the limited number of recorded flowering individuals. Its taxonomic identity became evident following the observation of flowering plants during field surveys conducted in 2025. *Puya ukumari* resembles *P. lineata* in possessing a simple, nodding, dark-colored inflorescence; however, it is readily distinguished by its considerably larger flowering plant height (160–200 cm vs. 40–80 cm), significantly wider inflorescence diameter (11–12.5 cm vs. 3.5–6 cm), larger lanate sepals (30–33 × 15–18 mm vs. 13–18 × 6–10 mm and floccose), and markedly darker, nearly black petals. These characters support its recognition as a distinct taxon within the group of *Puya* species with simple inflorescences. The species is currently known from a limited number of confirmed localities within a restricted elevational range. Based on its distribution and the observed threats to its habitat, a preliminary conservation assessment suggests that it should be classified as Endangered (EN) according to IUCN Red List criteria.

## Abstract (es)

Los bosques montanos de los Andes Tropicales se encuentran entre los ecosistemas con mayor diversidad y endemismo del mundo; sin embargo, aún permanecen insuficientemente documentados, especialmente en paisajes remotos y de compleja topografía. En este trabajo describimos *Puya ukumari***sp. nov**. (Bromeliaceae), una nueva especie altoandina procedente de los valles de Kosñipata y Mapacho, en el sureste del Perú, distribuida entre aproximadamente 2,300 y 3,300 m s.n.m. La especie fue encontrada durante un programa de monitoreo ecológico de largo plazo en la región, pero permaneció sin ser reconocida durante varios años debido a la limitada detectabilidad de los individuos en floración. Su identidad taxonómica se hizo evidente tras la observación de plantas florecidas durante evaluaciones de campo realizadas en 2025. *Puya ukumari* presenta semejanza superficial con *Puya lineata* al poseer una inflorescencia simple, péndula y de coloración oscura; sin embargo, se distingue fácilmente por una combinación de caracteres morfológicos, incluyendo una mayor altura de la planta en floración (160–200 cm vs. 40–80 cm), mayor diámetro de inflorescencia (11–12.5 cm vs. 3.5–6 cm), sépalos lanosos de mayor tamaño (30–33 × 15–18 mm vs. 13–18 × 6–10 mm y flocosos) y pétalos notablemente más oscuros, casi negros. Estos caracteres sustentan su reconocimiento como un taxón distinto dentro del grupo de especies de *Puya* con inflorescencia simple. *Puya ukumari* crece como planta terrestre en bosques montanos húmedos y hábitats ecotonales bosque–puna, formando parte del ensamblaje de bromeliáceas altoandinas. Actualmente, la especie se conoce de un número limitado de localidades confirmadas dentro de un rango altitudinal restringido. Con base en su distribución y las amenazas observadas sobre su hábitat, una evaluación preliminar de conservación sugiere su categorización como En Peligro (EN) según los criterios de la Lista Roja de la UICN.

## Introduction

Tropical Andean montane forests are among the most biodiverse and endemic-rich ecosystems worldwide, supporting exceptionally high plant diversity across steep elevational gradients and playing a critical role in regional climate regulation and ecosystem functioning (Myers et al. 2000; Malhi et al. 2010). However, these forests are also highly vulnerable to climate change and land-use transformation, as projected warming and shifts in precipitation regimes are expected to drive rapid upslope range shifts, habitat contraction, and the loss of suitable climatic conditions for many montane plant species (Feeley et al. 2011; Rapp and Silman 2012). The Kosñipata Valley in southeastern Peru constitutes a key elevational corridor within the Tropical Andes, linking Amazonian lowland forests with high-Andean ecosystems over short horizontal distances and encompassing a mosaic of cloud forests and ecotonal habitats that have been widely used to study patterns of plant diversity and turnover along elevation (Malhi et al. 2010; Feeley et al. 2011). Despite its recognized ecological importance, large portions of the montane flora of this region remain incompletely documented, particularly for taxa with restricted distributions or those occurring in remote and topographically complex landscapes, underscoring the need for continued botanical exploration and long-term field studies (Farfan-Rios et al. 2015).

The genus *Puya* Molina (Bromeliaceae, Puyoideae) represents one of the most diverse and morphologically complex lineages of bromeliads in South America (Smith 1952; Smith and Downs 1974; Givnish et al. 2007; Givnish et al. 2011; Manzanares 2020). Since the classical treatments by Mez (1896) and the comprehensive revision by Smith and Downs (1974), the number of recognized species has increased steadily, reaching approximately 230 taxa in recent compilations (Gouda and Butcher 2026 [continuously updated]; Jabaily and Sytsma 2013). *Puya* is distributed from Costa Rica to Chile and Argentina and occupies an exceptionally broad elevational range, from sea level to nearly 5,000 m, reflecting its ecological plasticity and diversification in montane environments (Smith and Downs 1974; Manzanares 2020). The radiation of the genus has been linked to Andean uplift and associated ecological shifts, which likely promoted rapid speciation and morphological diversification in high-elevation habitats (Givnish et al. 2011; Jabaily and Sytsma 2013; Aguirre-Santoro et al. 2024).

Despite its diversity, taxonomic work in *Puya* remains challenging (Zizka et al. 2013; Manzanares 2020). Infrageneric classifications have traditionally relied on inflorescence architecture, particularly the distinction between species with simple inflorescences and those with branched inflorescences bearing sterile apices (Mez 1896; Smith and Downs 1974). However, both morphological and molecular phylogenetic studies have demonstrated substantial homoplasy in these characters, indicating that several traditional groupings do not correspond strictly to monophyletic lineages, although they remain useful as functional taxonomic frameworks (Jabaily and Sytsma 2013; Givnish et al. 2014). In Peru, recent botanical exploration has led to the description of new Puya species and range extensions, yet significant gaps persist, especially in montane regions of the southeastern Andes, where incomplete sampling, rugged terrain, and limited accessibility continue to constrain taxonomic resolution (Janeba 2017; Treviño-Zevallos et al. 2019, 2025; Ayasta et al. 2021).

The discovery of *Puya ukumari* sp. nov. highlights the challenges of documenting plant diversity in tropical montane forests, where many species occur at low densities and in highly inaccessible habitats. In the Kosñipata Valley, terrestrial *Puya* species are common components of montane vegetation and are often identified based on vegetative traits alone when reproductive material is scarce or difficult to access, which can obscure species-level diversity among morphologically similar taxa. As a result, populations may remain undetected or misidentified for extended periods until flowering individuals are observed during targeted or repeated field surveys, underscoring the importance of sustained field presence for accurate taxonomic assessment in montane ecosystems.

In this study, we provide a formal description of *Puya ukumari*, including detailed morphological characterization, information on its distribution, habitat, and phenology, and a preliminary assessment of its conservation status following IUCN Red List criteria.

## Materials and methods

### Field survey and specimen collection

Field observations were conducted between 2022 and 2025 across an elevational range of approximately 700–3,800 m in the Kosñipata Valley, southeastern Peru, as part of long-term ecological monitoring programs based at Wayqecha Biological Station (13°10'29.8"S, 71°35'13.8"W) and Manu Biological Station (12°53'42.0"S, 71°24'13.4"W), two permanent field research platforms supporting biodiversity studies in Andean montane ecosystems.

In 2022, during fieldwork associated with dietary studies of collared Andean bears conducted within this monitoring framework, a terrestrial *Puya* bearing an inflorescence was observed and recognized as morphologically distinct from the sympatric and locally abundant *P. membranacea* L.B.Sm. However, the limited number of flowering individuals detected at that time, together with the species’ occurrence in highly inaccessible terrain, precluded immediate taxonomic follow-up.

In early 2025, tracking data from collared bears revealed repeated use of areas dominated by extensive patches of terrestrial bromeliads. Subsequent inspections of these areas revealed consistent vegetative differences between these plants and *P. membranacea*, particularly in leaf morphology. Seeds were collected in April 2025 from individuals located along the Erickson Trail (Manu National Park), while the first flowering individual attributable to the new species was documented in August 2025 in the community of Sunchubamba. This represented the first voucher specimen collected, bearing inflorescences with partially open flowers. Additional flowering individuals were recorded in the following months, allowing more complete documentation of reproductive characters.

Floral visitation was documented between 2025 and February 2026 through direct field observations and camera trap records at Wayqecha Biological Station.

## Taxonomic treatment

### Puya ukumari

Taxon classification

Plantae

PoalesBromeliaceae

Treviño, Yuca & Gouda sp. nov.

A3137EED-7F35-569C-8493-53B836FE0693

urn:lsid:ipni.org:names:77412419-1

**Figure 1.**
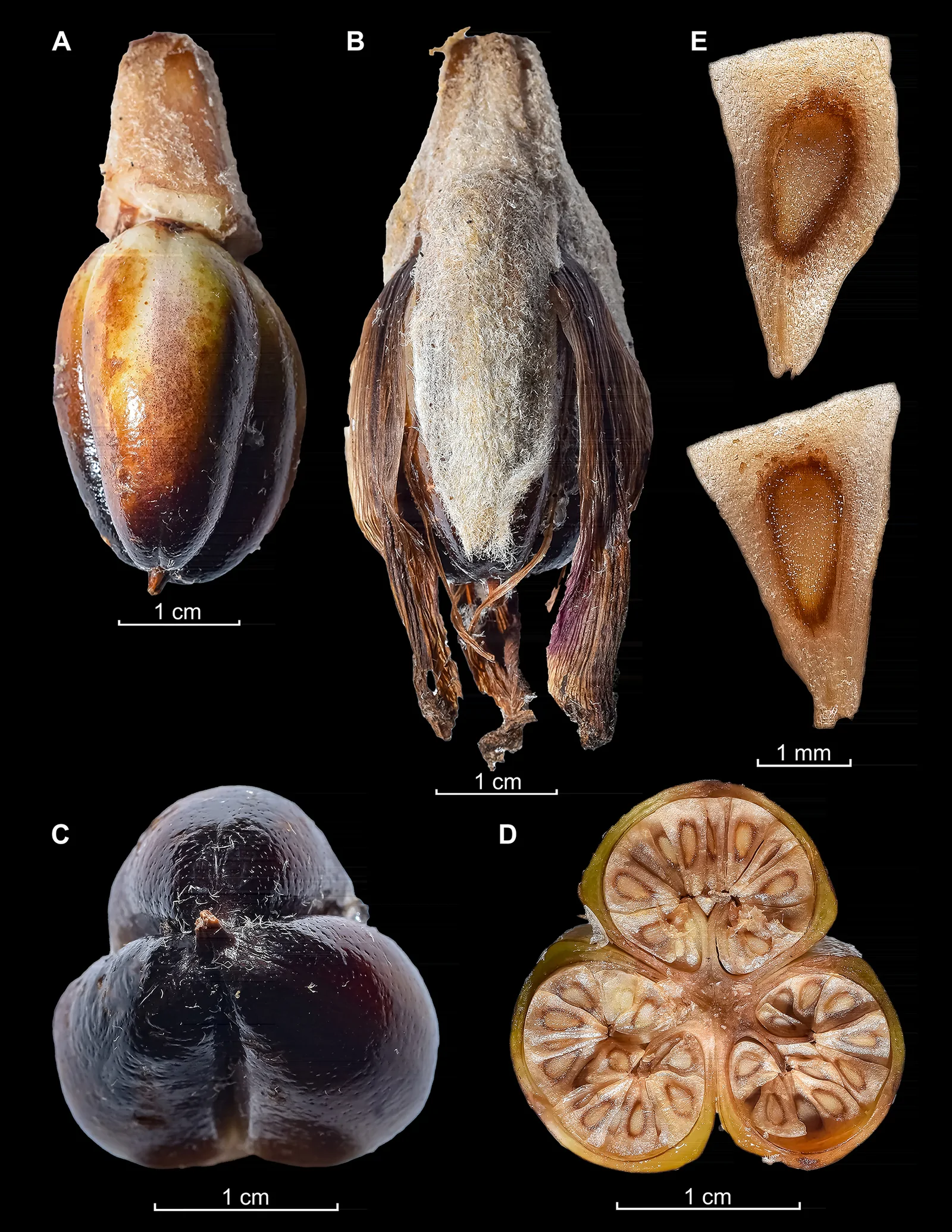
*Puya ukumari* sp. nov. **A**. Lateral view of the pedicel with a mature fruit; **B**. Fruit with the persistent calyx and corolla; **C**. Bottom view of the fruit; **D**. Transverse section of the fruit; **E**. Winged seeds. Photographs by Jhon Yuca.

**Figure 2.**
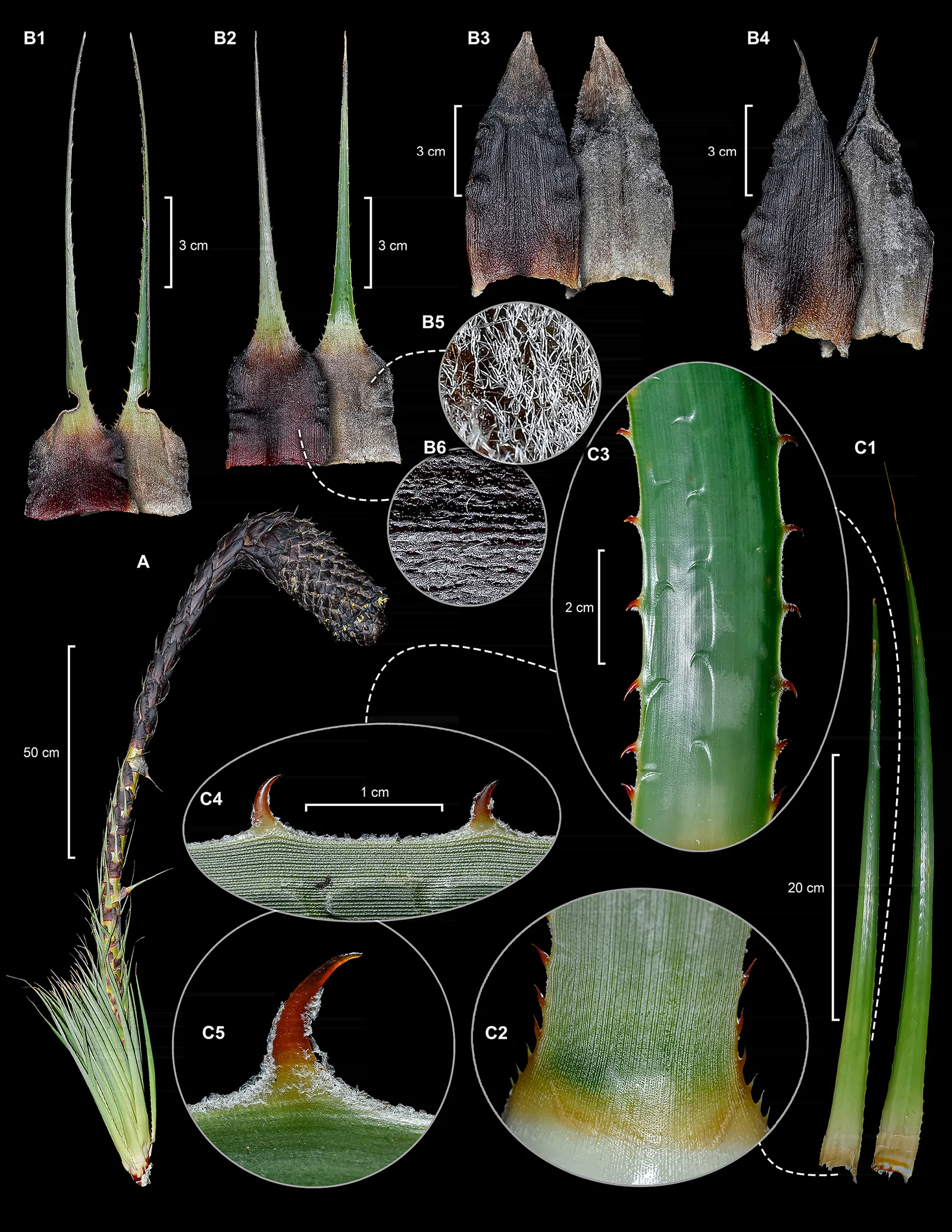
*Puya ukumari* sp. nov. **A**. Habit; **B1**. Basal inflorescence bracts; **B2**. Middle inflorescence bracts; **B3**. Upper inflorescence bracts; **B4**. Upper inflorescence bracts near the flowers; **B5**. Abaxial surface of the inflorescence bracts; **B6**. Adaxial surface of the inflorescence bracts; **C1**. Leaves adjacent to the peduncle; **C2**. Middle portion of the leaf blade; **C3**. Basal portion of the leaf; **C4**. Abaxial indument and marginal spines of the leaf; **C5**. Spine. Photographs by Jhon Yuca.

**Figure 3.**
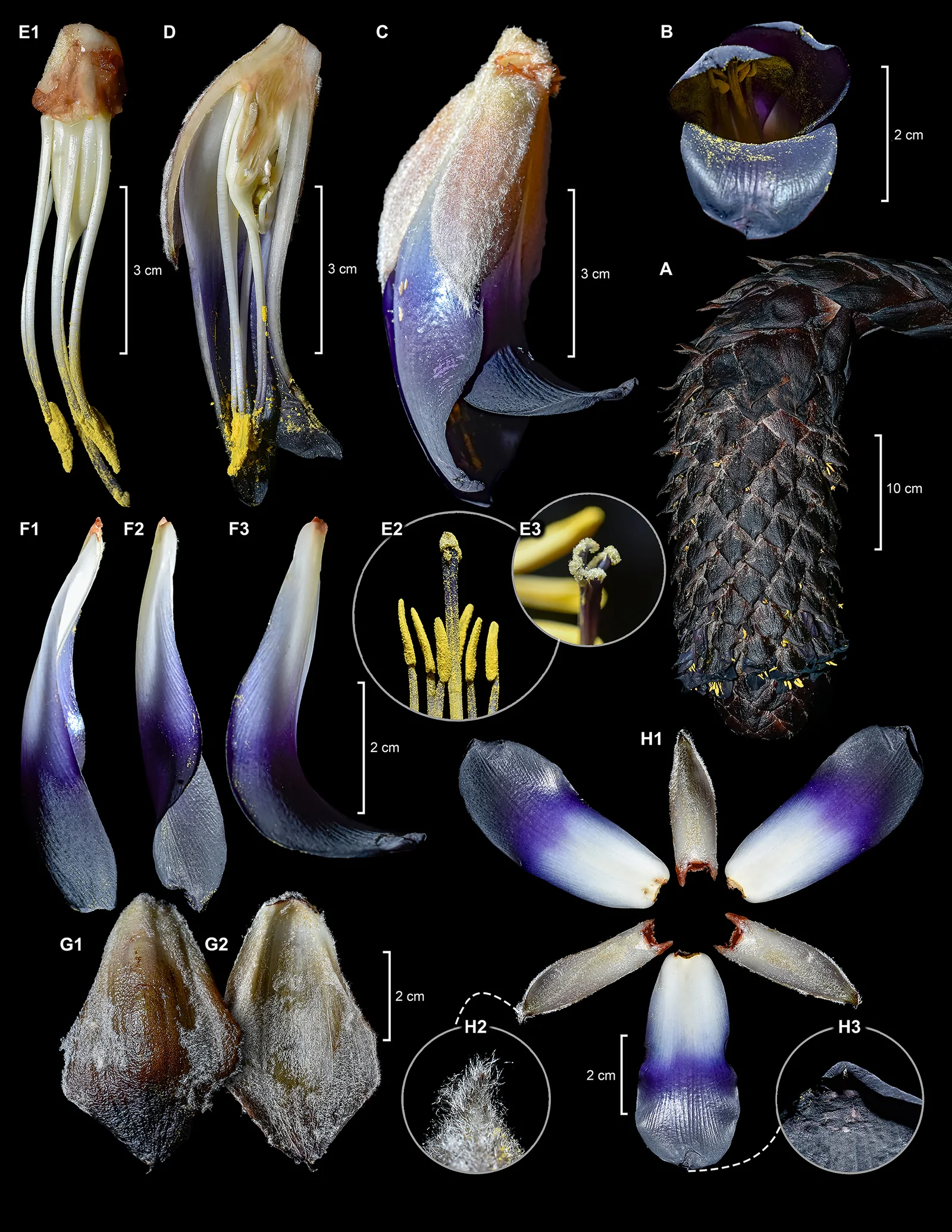
*Puya ukumari* sp. nov. **A**. Inflorescence; **B**. Frontal view of the flower; **C**. Lateral view of the flower; **D**. Longitudinal section of the flower; **E1**. Androecium and gynoecium; **E2**. Dorsifixed anthers; **E3**. Stigma with spirally twisted lobes; **F1**. Upper right petal; **F2**. Upper left petal; **F3**. Lower petal; **G1**. Adaxial view of the floral bract; **G2**. Abaxial view of the floral bract; **H1**. Perianth dissection, showing sepals and petals; **H2**. Sepal apex; **H3**. Petal apex. Photographs by Jhon Yuca.

**Figure 4.**
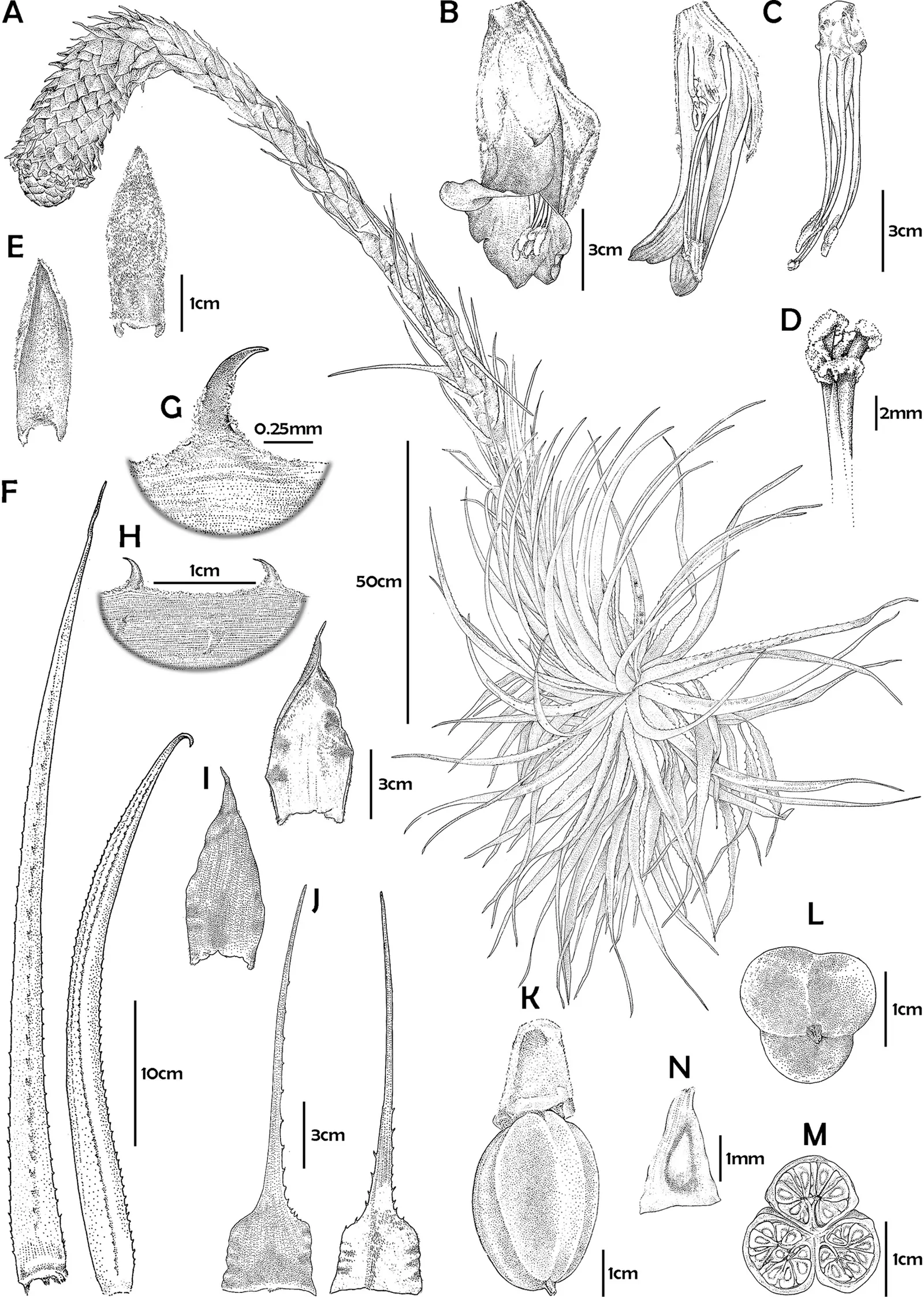
*Puya ukumari* sp. nov. **A**. Habit; **B**. Lateral view of the flower and longitudinal section of the flower; **C**. Androecium and gynoecium; **D**. Stigma with spirally twisted lobes; **E**. Adaxial and abaxial views of the floral bract; **F**. Juvenile leaves; **G**. Spine; **H**. Blade indument and marginal leaf spines; **I**. Upper inflorescence bracts near the flowers; **J**. Basal inflorescence bracts; **K**. Lateral view of the fruit; **L**. Bottom view of the fruit; **M**. Transverse section of the fruit; **N**. Winged seed. Illustration by Nidia Sanchez.

**Figure 5.**
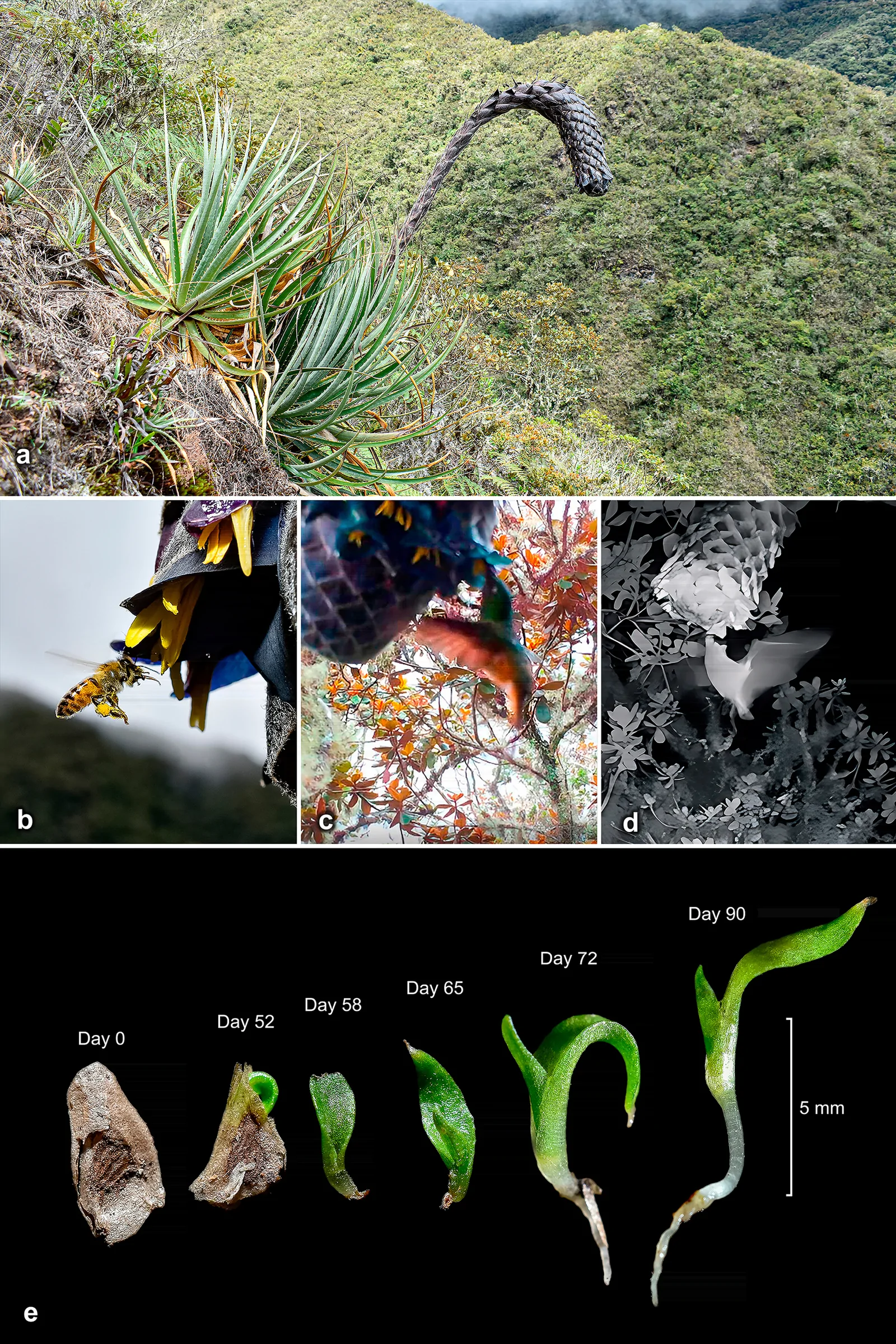
Ecological context, floral visitors, and early regeneration of *Puya ukumari* sp. nov. **a**. Habitat in humid upper montane forest and forest–puna ecotone, showing rocky slopes and discontinuous canopy where the species occurs; **b**. Diurnal floral visitor (bee, *Apis mellifera*) interacting with flowers during anthesis; **c**. Diurnal floral visitor *Boissonneaua matthewsii* approaching the inflorescence; **d**. Nocturnal floral visitor (*Anoura* sp.) recorded by camera trap during night activity; **e**. Seed germination and early seedling development under ambient montane forest conditions at Wayqecha Biological Station. Scale bar: 5 mm.

**Figure 6.**
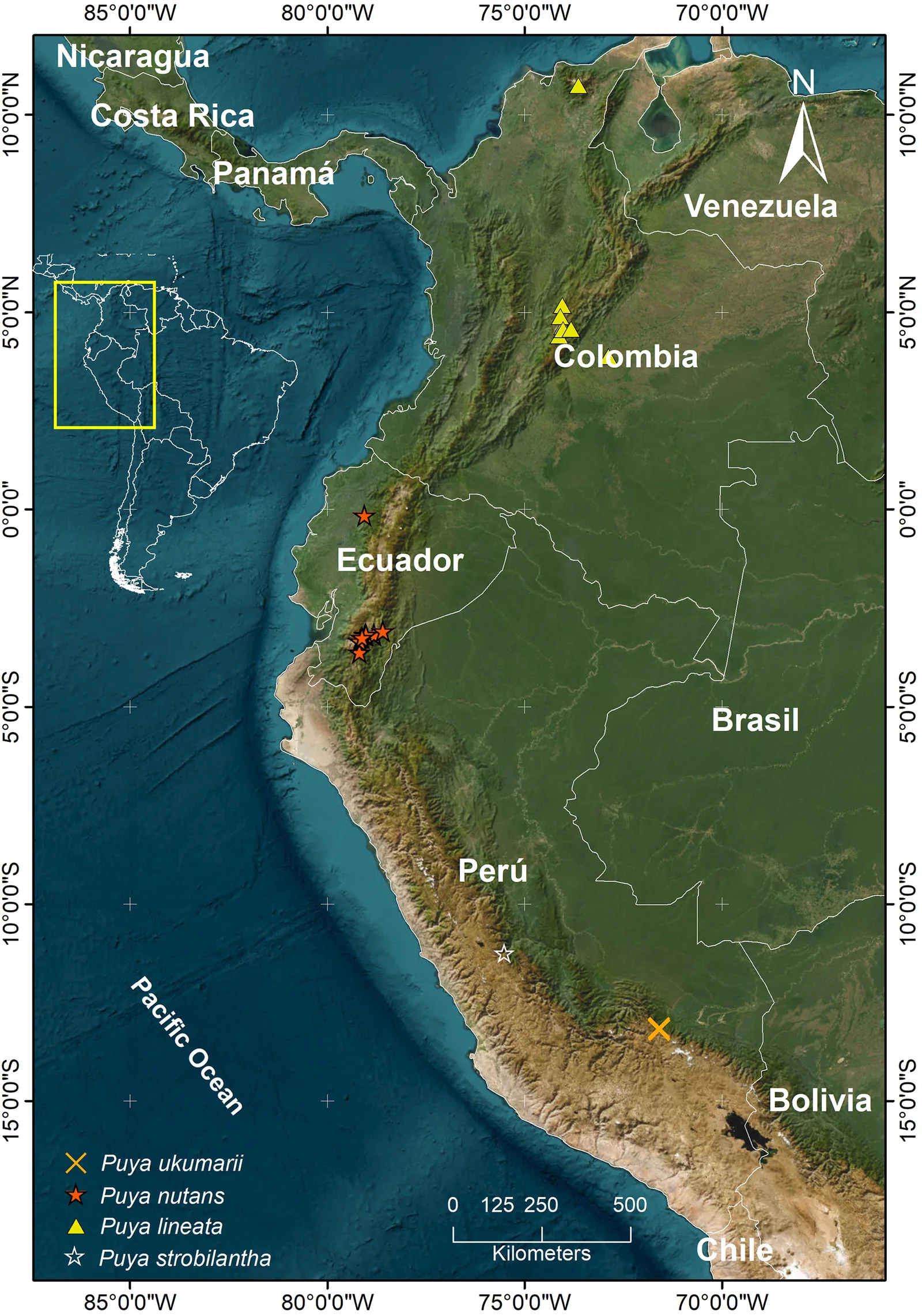
Geographic distribution of *Puya ukumari*, *P. nutans*, *P. lineata* and *P. strobilantha* in the northern and central Andes of South America.

Figs 1, 2, 3, 4, 5, 6

#### Diagnosis.

*Puya ukumari* is similar to *P. lineata* but is readily distinguished by its much larger flowering size (160–200 cm vs. 40–80 cm), significantly wider inflorescence (11–12.5 cm in diameter vs. 3.5–6 cm), and much larger sepals (30–33 × 15–18 mm vs. 13–18 × 6–10 mm), which are lanate in *P. ukumari* and floccose in *P. lineata*, as well as by its markedly darker petals (nearly black vs. blue).

#### Type.

Peru • Cusco: Province of Paucartambo, Trocha Sunchubamba, Juan Velasco Alvarado community, near the boundary of the Wayqecha Private Conservation Area, upper montane forest, 2907 m a.s.l., 02 Sep 2025, 13°11'57.8"S, 71°35'11.9"W, *J. Yuca & M. Mendoza JYP-146* (holotype: HSP; isotype: CUZ).

#### Description.

Perennial plant, flowering (126–)160–200(–235) cm tall, polycarpic, with three to seven rosettes per clone, rosettes 70–120 cm in diameter and 75–90 cm high. Leaves coriaceous, numerous; sheath 4.5–6 cm long, 5–6.5 cm wide, margins in the upper part serrate, adaxially mostly glabrous at the transition zone with the blade, abaxially lepidote; blade (45–)50–70(–75) cm long, 1.8–2.1 cm wide, very narrowly triangular, margins serrate near the base; spines 2–4 mm long, antrorsely or retrorsely serrulate near the basal fourth of the leaf, antrorsely serrulate toward the apex; apex sharp and pungent; adaxially glabrous, abaxially densely lepidote. Inflorescence 23–30 cm long, 11–12.5 cm wide, nutant, simple, blackish, racemose, strobiliform; peduncle (100–)110–150(–172) cm long, 39–41 mm in diameter, lepidote, with white stellate hairs easily detachable at maturity; peduncle bracts erect, the lower ones foliaceous, distal bracts 9.2–10.5 cm long, 3–4 cm wide, lanceolate, margins laxly serrulate, apex long-acuminate, lepidote on both surfaces, more densely so adaxially, blackish; axis 17–20 mm in diameter; floral bracts 65–80 mm long, 35–45 mm wide, obovate, acuminate, thin, margins entire, exceeding the sepals at anthesis, deciduous, lepidote on both surfaces, denser abaxially. Flowers pedicellate, 0.7–1.1 cm apart; pedicel (5–)7–10 mm long, 5–7 mm in diameter; sepals oblong, 30–33 mm long, 15–18 mm wide, thin, ecarinate but with a thickened midline, margins entire, apex acute or mucronate, densely white-lanate on both surfaces, white to bluish-gray with greenish tones toward the apex; petals 6–6.3 cm long, 22–25 mm wide, narrowly elliptic, obtuse, margins entire, glabrous, bluish to nearly black, whitish toward the base. Stamens and pistil visible, equal to or slightly shorter than the petals; filaments 4.7–5 cm long, 1–1.8 mm wide; anthers bilobed at the base, 12–13 mm long, 2–2.4 mm in diameter, dorsifixed at about one-fifth from the base; pollen yellow. Pistil 5.7–6 cm long, slightly shorter than the stamens; ovary subpyriform, superior, 16–18 mm long, 6–8 mm in diameter, whitish, glabrous; style 39–41 mm long, white, slender; stigma bluish-black, lobes conduplicate and spirally twisted. Fruit ellipsoid, 2.7–3.1 × 1.9–2.3 cm, shortly beaked; seeds triangular, winged, 2.3–3 mm long, 1.5–2 mm wide.

#### Etymology.

The specific epithet ukumari is a noun in apposition derived from the Quechua word “ukumari”, meaning Andean bear (*Tremarctos ornatus*). The species is dedicated to the Andean bear in recognition of the ecological importance of *Puya* species as a key component of its diet and of their close association within Andean montane ecosystems.

#### Distribution and ecology.

*Puya ukumari* is currently known from the southeastern Andes of Peru, where it inhabits humid montane forest ecosystems characterized by steep, often unstable terrain. Based on available collections and direct field observations, the species is currently known only from this region and exhibits a geographically restricted distribution. Confirmed records are distributed along the Kosñipata and Mapacho valleys in southern Peru, including Wayqecha Biological Station (Trocha Mariposa), Manu National Park (Erickson Trail), the Pillahuata sector, and nearby communities such as Juan Velasco Alvarado and Acobamba. These records span an elevational range between approximately 2300 and 3300 m a.s.l., representing the range currently documented for the species. The species may occur at lower or higher elevations within the same landscape, but additional field surveys are required to determine its full elevational distribution.

*Puya ukumari* occurs in habitats with a discontinuous canopy, low densities of accompanying vascular plants, and substrates dominated by rock outcrops and shallow soils. Individuals typically grow solitarily or in small, scattered groups of three to seven plants, often in highly inaccessible areas where topography limits both detection and collection and may contribute to the species’ apparent rarity. The species is associated with a diverse assemblage of vascular plants typical of Andean montane ecosystems. The arboreal stratum is composed mainly of small trees belonging to Proteaceae (*Oreocallis grandiflora* (Lam.) R. Br.), Cunoniaceae (*Weinmannia crassifolia* Ruiz & Pav.), Rosaceae (*Hesperomeles ferruginea* (Pers.) Benth.), Loranthaceae (*Gaiadendron punctatum* (Ruiz & Pav.) G. Don), Clusiaceae (*Clusia* L.), Escalloniaceae (*Escallonia paniculata* (Ruiz & Pav.) Roem. & Schult.), Myricaceae (*Morella pubescens* (Humb. & Bonpl. ex Willd.) Wilbur), and Clethraceae (*Clethra ferruginea* (Ruiz & Pav.) Link ex Spreng.).

The shrub and subshrub layer is dominated by Ericaceae (*Bejaria aestuans* Mutis, *Gaultheria reticulata* Kunth, *Gaultheria erecta* Vent., *Thibaudia diphylla* (Cav.) Hieron., *Cavendishia bracteata* (Ruiz & Pav. ex J. St.-Hil.) Hoerold, *Diogenesia vargasiana* Sleumer), Fabaceae (*Collaea speciosa* (Loisel.) DC.), Asteraceae (*Baccharis latifolia* (Ruiz & Pav.) Pers.), Melastomataceae (*Brachyotum* Triana, *Miconia* Ruiz & Pav.), and Myrtaceae (*Myrteola phylicoides* (Benth.) Landrum). The herbaceous stratum includes representatives of Orchidaceae (*Epidendrum secundum* Jacq., *Elleanthus conifer* (Rchb.f. & Warsz.) Rchb.f.), Poaceae (*Cortaderia nitida* (Kunth) Pilg., *Chusquea* Kunth), Dennstaedtiaceae (*Pteridium arachnoideum* (Kaulf.) Maxon), and Orobanchaceae (*Agalinis lanceolata* (Ruiz & Pav.) D’Arcy).

Flowering has been recorded between July and February, whereas fruiting individuals have been observed from January to June, indicating a clear association between reproductive phenology and local climatic seasonality. During the flowering period, frequent visits by bees, including *Apis mellifera*, were observed under daylight conditions. In addition, camera trap records documented visits by the hummingbird *Boissonneaua matthewsii* and bats *Anoura* sp. to the flowers. These observations indicate that *Puya ukumari* is visited by multiple vertebrate and invertebrate taxa, suggesting a diverse assemblage of floral visitors, consistent with previous studies on pollination in high-Andean bromeliads (Salinas et al. 2007). Although effective pollination was not experimentally confirmed, repeated visitation across taxa indicates that both insects and vertebrates may contribute to its reproductive ecology.

Under ambient montane forest conditions, where mean annual temperatures are approximately 12 ± 5 °C, seeds exhibit a dormancy period of about 30 days. Germination begins around 50 days after sowing, marked by seedling emergence, and early development is slow, with seedlings reaching the two-true-leaf stage after approximately 90 days. Germination observations were conducted at Wayqecha Biological Station (ca. 2900 m a.s.l.), within the natural distribution range of the species, in concordance with previous observations in high-Andean bromeliads (Hornung-Leoni and Sosa 2008) (Fig. 5).

#### Notes.

*Puya ukumari* resembles *P. lineata* Mez in the nodding, simple, sparsely lepidote, blackish inflorescence, but it is readily distinguished by its larger flowering size, wider inflorescence, and darker petals (see Diagnosis and Table 1). *Puya lineata* does not always have a nodding inflorescence and may also present erect inflorescences. *Puya nutans* L.B.Sm. also has a nodding inflorescence and could be confused with the new species, but *P. ukumari* differs by its much larger flowering size and by the floral bracts, which are sparsely white-floccose and blackish, versus the very dense ferruginous-brown indumentum reported for *P. nutans*. A specimen from Cusco previously identified as *P. nutans* (Cano 4324) does not correspond to this species, supporting the current view that *P. nutans* is restricted to Ecuador. *Puya strobilantha* Mez is also superficially similar to *P. ukumari*, particularly in the compact, strobiliform appearance of the inflorescence; however, *P. ukumari* differs by its larger inflorescence (23–30 × 11–12.5 cm vs. ca. 14 × 6 cm), larger, obovate and acuminate floral bracts (65–80 × 35–45 mm vs. ca. 40 × 30 mm, very broadly ovate and acute), and longer, thin, densely lanate sepals (30–33 mm vs. ca. 25 mm, very thick, rigid, and araneous-tomentellous).

**Table 1.** Morphological comparison of *Puya ukumari* sp. nov. with the morphologically similar species *Puya lineata* Mez, *Puya nutans* L.B.Sm. and *Puya strobilantha* Mez.

**Character**	***Puya ukumari***	***Puya lineata***	***Puya nutans***	***Puya strobilantha***
**Plant**				
Flowering size	160–200 cm	40–80 cm	60–90 cm	ca. 100 cm
**Leaf sheath**				
Length	4.5–6 cm	ca. 4 cm	ca. 10 cm	–
Width	5–6.5 cm	–	ca. 10 cm	–
Margins	Serrate in upper part	–	Entire	–
Indumentum (adaxial surface)	Glabrous	–	Lepidote	–
**Leaf blade**				
Length	(45–)50–70(–75) cm	30–60 cm	20–35 cm	up to 90 cm
Width	1.8–2.1 cm	0.8–2.2 cm	ca. 1.5 cm	2.5 cm
**Inflorescence**				
Diameter	11–12.5 cm	3.5–6 cm	ca. 9 cm	ca. 6 cm
**Peduncle**				
Length	110–150 cm	–	50–60 cm	–
Diameter	39–41 mm	–	ca. 15 mm	–
**Peduncle bracts (upper)**				
Width	3–4 cm	–	ca. 5 cm	–
Form	Lanceolate	Broadly ovate	Elliptic	Very broadly ovate-elliptic
Apex	Acuminate	Apiculate	Acuminate	Abruptly acute
**Floral bracts**				
Length	65–80 mm	–	ca. 35 mm	40 mm
Width	35–45 mm	–	ca. 25 mm	30 mm
Form	Obovate	–	Ovate	Very broadly ovate
Apex	Acuminate	–	Acute to apiculate	Acute
Margins	Entire	–	Denticulate	Entire
**Sepals**				
Form	Oblong	Oblong	Obovate	Oblong-elliptic
Length	30–33 mm	13–18 mm	ca. 18 mm	ca. 25 mm
Width	15–18 mm	6–10 mm	ca. 15 mm	–
Structure	Thin	–	Coriaceous, rugose	Very thick and rigid
Apex	Acute or mucronate	Apiculate	Obtuse	Rounded and mucronulate
Indumentum (adaxial surface)	Lanate	Floccose	–	Araneous-tomentellous
**Petals**				
Length	6–6.3 cm	3–3.5 cm	ca. 4 cm	–
Color	Bluish-black	Blue	–	–

In addition to the morphological differences outlined above, geographic patterns further support the separation of these taxa. As shown in Fig. 6, *P. ukumari* is currently known from a limited number of confirmed localities in the southeastern Andes of Peru (Cusco). In contrast, *P. lineata* exhibits a clearly disjunct and more northerly distribution, occurring in the northern Andes of South America, primarily in Colombia and Venezuela, with no confirmed records from Peru. *Puya nutans* is likewise geographically isolated, being restricted to the Ecuadorian Andes and showing no known spatial overlap with *P. ukumari* (GBIF.org 2025). *Puya strobilantha*, although also known from Peru, was described from the vicinity of Huacapistana near Palca, Tarma, Junín, in the central Peruvian Andes at 2000–2100 m (Smith and Downs 1974), geographically separated from known populations of *P. ukumari* in Cusco. This pronounced geographic disjunction and the lack of documented distributional continuity among the three species further support the recognition of *P. ukumari* as a distinct species, separate from *P. lineata*, *P. nutans* and *P. strobilantha*.

#### Conservation status.

Following the IUCN Red List Categories and Criteria (IUCN 2012), we propose a preliminary Endangered (EN) categorization for *P. ukumari* under criteria B1ab(iii)+2ab(iii). This recommendation has not yet been formally submitted to the IUCN Red List and should be considered provisional pending a complete assessment. The species is currently known from five locations (*sensu* IUCN) in the southeastern Andes of Peru, distributed across the Kosñipata and Mapacho valleys, including Acobamba (Mapacho Valley), Sunchubamba, Wayqecha, Pillahuata, and Manu National Park. Based on available georeferenced records, the extent of occurrence (EOO) is estimated at 33.16 km^2^, and the area of occupancy (AOO) is estimated at 24 km^2^, calculated using the standard 2 × 2 km IUCN grid. These values are considerably below the thresholds established for Endangered under criteria B1 and B2.

Although suitable habitats may extend beyond the currently documented range, *P. ukumari* is known from a restricted number of confirmed records and occurs in fragmented and sparsely distributed populations. Populations occur mainly in montane forest habitats and ecotonal areas, where habitat degradation, livestock activities, fire, and landslides represent ongoing or potential threats. The population from Acobamba is particularly exposed to habitat disturbance due to its proximity to roads, whereas other populations occur in more remote and difficult-to-access areas. Some populations in Sunchubamba are affected by livestock activities, in concordance with recent reports on anthropogenic impacts in montane ecosystems of Cusco (Huaman-Arqque et al. 2022). Conversely, populations within Wayqecha Biological Station and Manu National Park benefit from conservation protection; however, their restricted geographic range and the potential impacts of stochastic events and climate-related changes remain relevant concerns. Considering the limited distribution (EOO = 33.16 km^2^; AOO = 24 km^2^), the small number of known locations, and the continuing decline in habitat quality, we recommend a preliminary categorization of *P. ukumari* as Endangered (EN) under B1ab(iii)+2ab(iii).

##### Key to the species of *Puya* with simple strobiliform inflorescences

This key was adapted from the “Encyclopaedia of Bromeliads” (Gouda and Butcher 2026 [continuously updated]), which provides an updated version of the keys originally derived from Smith and Downs’ treatment of *Puya* in Flora Neotropica. Only species with simple inflorescences were included, excluding taxa with compound inflorescences or fasciculate flowers. *Puya ukumari* is added here based on the morphological evidence presented in this study.

**Table:** 

1	Floral bracts conspicuously serrate	**2**
–	Floral bracts entire or very obscurely serrulate	**9**
2	Apices of floral bracts reflexed	**3**
–	Apices of floral bracts erect	**4**
3	Bracts coriaceous (Peru)	***Puya coriacea***
–	Bracts thin; inflorescence sparsely white-lanate; (Argentina)	***Puya weberiana***
4	Flowers distinctly pedicellate	**5**
–	Flowers sessile or subsessile	**8**
5	Flowering plant 4–10 dm high	**6**
–	Flowering plant 1.5–3 dm high	**7**
6	Bracts finely pectinate-serrate; sepals 13–18 mm long (Colombia–Venezuela)	***Puya lineata***
–	Bracts coarsely serrate with broad flat teeth; sepals 20–25 mm long (Peru)	***Puya angulonis***
7	Floral bracts serrulate; sepals 20–30 mm long, oblong-lanceolate, acute (Argentina)	***Puya volcanensis***
–	Floral bracts pectinate-serrate; sepals 17–20 mm long, elliptic, obtuse (Peru)	***Puya pratensis***
8	Inflorescence rufous-lanate; sepals 18–30 mm long (Colombia)	***Puya vestita***
–	Inflorescence glabrous except for the furfuraceous axis; sepals 12 mm long (Ecuador)	***Puya eryngioides***
9	Apical part or all of each outer bract reflexed	**10**
–	Apical parts of outer bracts erect	**22**
10	Flowers and bracts reflexed; sepals narrowly spatulate, obtuse, 40 mm long (Peru)	***Puya huancavelicae***
–	Flowers erect to spreading; only the apices of bracts reflexed	**11**
11	Floral bracts narrow, leaving much of flower exposed	**12**
–	Floral bracts ample, ovate, elliptic or triangular	**14**
12	Pedicels obconic, very short (Peru)	***Puya lanata***
–	Pedicels rather slender, 12–15 mm long	**13**
13	Flowers arcuate-spreading (Colombia)	***Puya sanctae-martae***
–	Flowers erect or suberect (Bolivia)	***Puya kuntzeana***
14	Flowering plant 3–4 m high; floral bracts 10–14 cm long (Argentina)	***Puya yakespala***
–	Flowering plant and floral bracts much smaller	**15**
15	Leaf blades entire along their apical quarter (Colombia)	***Puya nivalis***
–	Leaf blades serrate throughout, sometimes more laxly so toward apex	**16**
16	Floral bracts much shorter than sepals (Peru)	***Puya lanuginosa***
–	Floral bracts covering sepals	**17**
17	Sepals 14 mm long, acuminate (Bolivia)	***Puya tunarensis***
–	Sepals 20–40 mm long, acuminate to broadly acute	**18**
18	Inner face of floral bracts glabrous and nearly black, contrasting sharply with pale persistent indument of outer face (Peru)	***Puya cerrateana***
–	Inner face of floral bracts almost exactly like outer face, not blackening	**19**
19	Sepals 35–37 mm long	**20**
–	Sepals 20–25 mm long	**21**
20	Floral bracts subcoriaceous, greenish (Peru)	***Puya mariae***
–	Floral bracts membranaceous, rose (Argentina)	***Puya weberiana***
21	Floral bracts recurved from near apex only (Peru)	***Puya isabellina***
–	Floral bracts more than half recurved (Colombia)	***Puya trianae***
22	Inflorescence nutant	**23**
–	Inflorescence erect or suberect	**28**
23	Upper peduncle-bracts and floral bracts firm, subchartaceous, opaque	**24**
–	Upper peduncle-bracts and floral bracts membranaceous, translucent (Venezuela)	***Puya venezuelana***
24	Leaf blades upright, up to 20 mm wide; floral bracts closely imbricate, margins not recurving	**25**
–	Leaf blades curving and hanging downward, over 20 mm wide; floral bracts divergent after anthesis, green and glabrescent (Colombia)	***Puya pendula***
25	Floral bracts ca. 30 mm long; sepals lanceolate, obtuse, ca. 17 mm long (Colombia)	***Puya dichroa***
–	Floral bracts 30–80 mm long; sepals narrowly oblong, obovate or oblong, 18–33 mm long	**26**
26	Floral bracts minutely but distinctly lepidote, pale rose to pale greenish-rose (Venezuela)	***Puya guaramacalana***
–	Floral bracts densely lepidote. Brown to blackish	**27**
27	Floral bracts ovate, acute to apiculate, denticulate, ca. 35 mm long; sepals obovate, ca. 18 mm long (Ecuador)	***Puya nutans***
–	Floral bracts obovate, acuminate, entire, 65–80 mm long; sepals oblong, 30–33 mm long (Peru)	***Puya ukumari***
28	Sepals thinly vestite to glabrous	**29**
–	Sepals densely lanate	**35**
29	Sepals 12–13 mm long; floral bracts acuminate (Bolivia)	***Puya humilis***
–	Sepals 15–33 mm long	**30**
30	Floral bracts narrow lanceolate, acuminate; sepals triangular-acute, 25 mm long (Bolivia)	***Puya kuntzeana***
–	Floral bracts broad	**31**
31	Leaf blades 7–8 mm wide; inflorescence few-flowered, 4–7 cm long (Colombia)	***Puya brachystachya***
–	Leaf blades 25–50 mm wide	**32**
32	Pedicels 7–15 mm long	**33**
–	Pedicels very short and stout, flowers appearing subsessile	**34**
33	Sepals narrowly rounded, 20–23 mm long (Colombia)	***Puya ochroleuca***
–	Sepals broadly lanceolate, 30–33 mm long (Peru)	***Puya dolichostrobila***
34	Inflorescence cylindric, 30 cm long, 10 cm in diameter (Peru)	***Puya isabellina***
–	Inflorescence ellipsoid, 14 cm long, 6 cm in diameter (Peru)	***Puya strobilantha***
35	Leaf blades densely lepidote above as well as beneath; sepals attenuate (Peru)	***Puya macrura***
–	Leaf blades soon glabrous above	**36**
36	Inflorescence over 15 cm long	**37**
–	Inflorescence 5–8 cm long; sepals obtuse	**40**
37	Sepals membranaceous, elliptic, 27 mm long; inflorescence slenderly cylindric, 4 cm in diameter (Colombia)	***Puya gargantae***
–	Sepals coriaceous, lance-oblong or narrowly elliptic, 22–28 mm long	**38**
38	Floral bracts soon glabrous and contrasting with remainder of inflorescence (Peru)	***Puya angusta***
–	Floral bracts equally vestite with remainder of inflorescence	**39**
39	Leaf blades sparsely lepidote beneath; sepals 22 mm long (Bolivia)	***Puya brittoniana***
–	Leaf blades densely appressed-lepidote beneath; sepals 28 mm long (Colombia)	***Puya alpicola***
40	Inflorescence globose; indument nearly white; sepals 20–28 mm long (Colombia)	***Puya santanderensis***
–	Inflorescence globose; indument ferruginous; sepals 15 mm long (Venezuela)	***Puya cardonae***

## Supplementary Material

XML Treatment for Puya ukumari

## Additional information

### Artificial Intelligence (AI) use

The authors accept full responsibility for the content of the manuscript, including the disclosure of any use of AI.

Regarding the use of AI in the preparation of this manuscript, the authors declare the following: GPT.

**Used for**: Language, style and writing.

ChatGPT (OpenAI) was used solely to assist with English grammar, language, style, structure, and clarity during manuscript preparation. The authors developed and verified all scientific content, interpretations, analyses, and conclusions and take full responsibility for the accuracy and integrity of the manuscript.

### Author ORCIDs

J.A. Yuca-Palomino https://orcid.org/0000-0002-2054-5927

N.G. Mamani-Mayta https://orcid.org/0000-0002-1949-6865

L.A. Champi-Charalla https://orcid.org/0009-0007-9485-8946

E. Condori-Cuti https://orcid.org/0009-0006-5012-5591

N. Llacta-Gomez https://orcid.org/0009-0005-1491-537X

I.F. Treviño-Zevallos https://orcid.org/0000-0002-2406-7862

E.J. Gouda https://orcid.org/0000-0003-3578-8072

A. Forsyth https://orcid.org/0000-0001-5567-7279

A. Whitworth https://orcid.org/0000-0001-6197-996X

R. Pillco-Huarcaya https://orcid.org/0000-0003-0068-3852

### Data availability

All of the data that support the findings of this study are available in the main text or Supplementary Information.

## Supplementary materials

Supplementary material 1 Geographic records Data type xlsx Explanation note Geographic records of *Puya ukumari* sp. nov. used for distribution analysis. https://binary.pensoft.net/file/1809992This dataset is made available under the Open Database License (http://opendatacommons.org/licenses/odbl/1.0/). The Open Database License (ODbL) is a license agreement intended to allow users to freely share, modify, and use this Dataset while maintaining this same freedom for others, provided that the original source and author(s) are credited. Jhon A. Yuca-Palomino, Luis A. Champi-Charalla, Narciso Llacta-Gomez
